# Decoupling Growth and Protein Production in CHO Cells: A Targeted Approach

**DOI:** 10.3389/fbioe.2021.658325

**Published:** 2021-06-02

**Authors:** James S. Donaldson, Matthew P. Dale, Susan J. Rosser

**Affiliations:** School of Biological Sciences, University of Edinburgh, Edinburgh, United Kingdom

**Keywords:** biomanufacturing, synthetic biology, decoupling production from growth, CRISPR/Cas9, CHO cell culture

## Abstract

Fed-batch cultures of Chinese Hamster Ovary cells have been used to produce high quantities of biotherapeutics, particularly monoclonal antibodies. However, a growing number of next-generation biotherapeutics, such as bi-specific antibodies and fusion proteins, are difficult to express using standard fed-batch processes. Decoupling cell growth and biotherapeutic production is becoming an increasingly desired strategy for the biomanufacturing industry, especially for difficult-to-express products. Cells are grown to a high cell density in the absence of recombinant protein production (the growth phase), then expression of the recombinant protein is induced and cell proliferation halted (the production phase), usually by combining an inducible gene expression system with a proliferation control strategy. Separating the growth and production phases allows cell resources to be more efficiently directed toward either growth or production, improving growth characteristics and enhancing the production of difficult to express proteins. However, current mammalian cell proliferation control methods rely on temperature shifts and chemical agents, which interact with many non-proliferation pathways, leading to variable impacts on product quality and culture viability. Synthetic biology offers an alternative approach by strategically targeting proliferation pathways to arrest cell growth but have largely remained unused in industrial bioproduction. Due to recent developments in microbial decoupling systems and advances in available mammalian cell engineering tools, we propose that the synthetic biology approach to decoupling growth and production needs revisiting.

## Introduction

Industrial-scale production of many biotherapeutics relies on the culture of Chinese Hamster Ovary (CHO) cells. In fed-batch cultures, nutrients are added to the culture to prevent nutrient limitation, prolong culture duration, and maximize product titer. Once culture viability drops below a certain threshold, the culture is harvested and the product is purified. Advances in cell engineering, expression vector design, and process development have allowed monoclonal antibody (mAb) titers to reach 10 g/L. However, high expression of recombinant genes places a large metabolic burden on the cell, which can result in the downregulation of recombinant gene expression. To avoid unstable cell lines entering the manufacturing stage, stability studies (which remain a significant bottleneck in cell line development timelines) are performed to identify cell lines that maintain high gene expression over the culture period. Additionally, a growing range of next-generation biotherapeutics (such as bi-specific antibodies, fusion proteins, and toxic proteins) are being produced as a result of recent developments in protein engineering. These are often difficult to express in CHO cells at desired quantities. For example, production of hyperactive human DNase I is particularly challenging with standard culture processes, because protein expression negatively impacts cell growth, viability, and stability (Lam et al., 2017).

An increasingly attractive method of production is to decouple the growth and production phases of the culture process, usually by combining an inducible gene expression system with a proliferation control strategy (Misaghi et al., 2014; Lam et al., 2017; Poulain et al., 2017). Under such a system, cells are initially grown up to high cell density in the absence of recombinant protein production (the growth phase). Once the desired cell density is reached, expression of the recombinant protein is induced and cell proliferation is halted (the production phase). Proliferation is usually arrested in the G1 phase of the cell cycle, which is associated with larger cells and increased ribosomal protein S6 levels (Carvalhal et al., 2003; Bi et al., 2004). Separating the growth and production phases allows cell resources to be more efficiently utilized in each phase, improving growth characteristics and enhancing production stability (Misaghi et al., 2014). It also avoids expression of cytotoxic recombinant proteins during the growth phrase, reducing the negative impact on cell growth and viability (Lam et al., 2017). Inducing global downregulation of translation (apart from the product gene) during the production phase can increase product purity to aid downstream processing (Bojar et al., 2019).

However, the benefits of decoupling growth and production in mammalian cells are challenged by suboptimal proliferation control strategies. Most commonly, cells are arrested by shifting the culture temperature from 37°C to 30–35°C, or through the addition of chemical agents [such as sodium butyrate (Hong et al., 2014)] to the culture. Both strategies successfully arrest cell growth, usually in the G1 phase, while improving specific productivity and reducing nutrient uptake and waste production (Marchant et al., 2008). However, both strategies also impact other non-proliferation pathways. Transcriptomic studies have shown that temperature shifts cause wide-scale changes in gene expression in the cell (Bedoya-López et al., 2016) and can impact certain product quality attributes (e.g., glycosylation profile; Nam et al., 2008). In addition to negatively impacting product quality (Hong et al., 2014), chemical agents such as sodium butyrate also trigger apoptotic pathways (Kim and Lee, 2000). Model-based approaches have been developed to reduce the number of experiments required to determine the optimum timing for the temperature shift, to maximize product titer (Paul et al., 2019). However, decoupling growth and production usually requires extensive and time-consuming process development work to maximize titer, while avoiding negative product quality consequences (Xu et al., 2019). Lack of a suitable targeted proliferation control approach hinders rapid development of a decoupled production process.

Recently, there have been significant advances in decoupling growth and production in microbial systems using synthetic biology approaches (Izard et al., 2015; Li et al., 2016; Durante-Rodríguez et al., 2018; Stargardt et al., 2020). However, it has been over 20 years since the overexpression of cyclin-dependent kinase inhibitors (p21^*Cip*1^, p27^*Kip*1^, and p53) was first used to decouple growth and production in mammalian cells (Fussenegger et al., 1997). Implementation of this strategy was limited by a lack of appropriate inducible gene expression systems and its weak impact on mammalian cell proliferation (Weber and Fussenegger, 2007). Since then, there have been significant advances in synthetic biology tool development, but their application in decoupling growth and production in mammalian cells has largely remained unexplored (Guha et al., 2017; Kallunki et al., 2019; Trauth et al., 2019). Here, we discuss the benefits of a targeted approach to proliferation control and discuss how recently developed tools in mammalian synthetic biology provide a promising solution for decoupling growth and production.

## Strategic Proliferation Control: Towards a Targeted Approach

**FIGURE 1:**
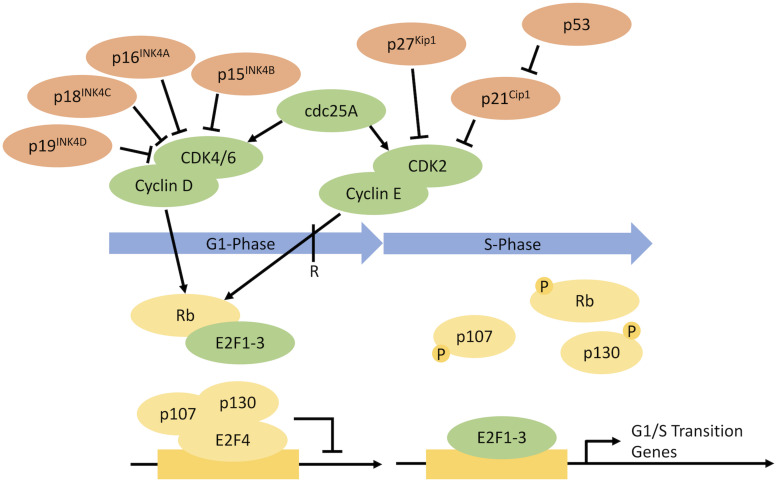
Regulation of the progression through G1 phase into S phase (Sunley and Butler, 2010). The progression through G1 into S phase is governed by a series of cell-cycle regulators, with some regulators promoting progression and others inhibiting progression. CKIs for potential overexpression are shown in red. Potential targets for gene knockdown/protein degradation are shown in green.

A targeted approach to cell proliferation control could hold the key to arresting cell proliferation at the G1 phase, the phase associated with larger cells and increased ribosomal protein S6 levels, while maintaining product quality. Progression through the cell cycle is governed by the regulated activation, degradation, and synthesis of a series of cell-cycle regulators. During G1, cells make a decision whether to irreversibly commit to a new round of cell division or remain in a non-proliferative G0 state (the restriction point). Progression past the restriction point is governed by the retinoblastoma (RB) protein, an inhibitor of G1/S gene transcription (Figure 1). Cyclin D-CDK4/6 and Cyclin E-CDK2 complexes phosphorylate RB, enabling the expression of G1/S genes. Cyclin-dependent kinase inhibitors (CKIs) regulate Cyclin-CDK activity (Alberts et al., 2002; Harper and Brooks, 2004).

The mechanism by which temperature shifts induce cell-cycle arrest is thought to involve membrane-dependent activation of the ataxia telangiectasia mutated and Rad3-related kinase (ATR)–p53–p21 pathway (Roobol et al., 2011). However, as previously mentioned, temperature shifts also cause wide-scale changes in gene expression in the cell (Bedoya-López et al., 2016) which can negatively impact product quality attributes. By contrast, targeted approaches to proliferation control should have far fewer off-target effects. Indeed, targeted inhibition of CDK4/6 activity using a highly selective cancer therapeutic molecule (PD 0332991) (Fry et al., 2004) increased specific mAb productivity and prolonged cell culture, while having minimal impact on product quality (Du et al., 2015). In addition, by contrast with temperature shift, targeted CDK4/6 inhibition did not lead to changes in expression of genes involved in the glycosylation pathway (Du et al., 2015; Bedoya-López et al., 2016). Thus, targeted inhibition approaches may avoid the negative impact from off-target effects of current cell proliferation control strategies for decoupling growth and production in mammalian cells. However, the high cost and physiological activity of cancer drugs limit their potential to be applied for such purposes in industrial bioproduction (Weber and Fussenegger, 2007). Synthetic biology strategies, which selectively target the activity of cell-cycle regulators through cell engineering approaches, could provide a less expensive and more industrially viable solution.

Previous synthetic biology strategies have focused on the overexpression of cyclin-dependent kinase inhibitors (CKIs), with p21^*Cip*1^, p53, and P27^*Kip*1^ the most studied (Fussenegger et al., 1997; Mazur et al., 1998; Carvalhal et al., 2003; Bi et al., 2004). The original approach controlled the expression of the CKI and product gene, on a dicistronic vector, with a doxycycline-inducible gene expression system (Fussenegger et al., 1997). Upon addition of doxycycline, cells were arrested in G1 and product expression was switched on, decoupling the growth and production phases of the culture process (Table 1A). Since then, multiple approaches have been tested, using a variety of inducible promoters and gene combinations (Mazur et al., 1998; Carvalhal et al., 2003; Bi et al., 2004). The overexpression of CKIs caused increases in specific productivity, but the degree of specific productivity increase was variable (likely due to the inherent variability in transfection methods available at the time and the expression systems used). Overexpression of p21^*Cip*1^ led to a fourfold increase in specific productivity in a stable mAb-producing CHO cell line but not in a stable secreted embryonic alkaline phosphatase (SEAP)-producing CHO cell line (Mazur et al., 1998; Bi et al., 2004). However, the implementation of CKI overexpression for cell proliferation control has been limited by its weak impact on proliferation. Combining overexpression of CKIs with a temperature shift showed that the temperature shift had the dominant impact on cell proliferation (Kaufmann et al., 2001). This strongly suggests that novel synthetic biology approaches are required for the manipulation of proliferation pathways.

**TABLE 1:** Mammalian synthetic biology tools which could be used for decoupling growth and production.

Method	Concept	Advantages	Disadvantages	Tested in CHO cells?
**(A)** Overexpression of CKIs	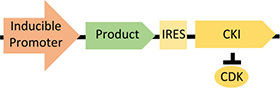	• Known increase in specific productivity	• Weak impact on proliferation	• Yes (Fussenegger et al., 1997; Mazur et al., 1998; Carvalhal et al., 2003; Bi et al., 2004)

**(B)** Caffeine-inducible dimerization of protein kinase R (Bojar et al., 2019)	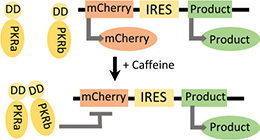	• Improved product purity • Caffeine as an inducer	• Limited product titer	• Yes—system tested in multiple industrially relevant cell lines including CHO cells (Bojar et al., 2019)

**(C)** Degradation of cell-cycle regulators (small-molecule induction)	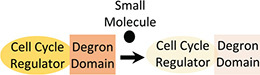	• Fast acting cell-cycle arrest	• Lack of industry appropriate small molecules • Multiple recombinant genes • Addition of degron can impact activity of POI	• Not yet used to target cell-cycle regulators in CHO cells • AID system previously tested in CHO cells (Kleinjan et al., 2017)

**(D)** Degradation of cell-cycle regulators (optogenetic approach)	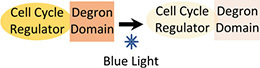	• Fast acting cell-cycle arrest • Light is non-toxic and can be instantly applied and removed	• Addition of degron can impact activity of POI	• Not yet used to target cell-cycle regulators in CHO cells • Dual-controlled “Blue-OFF” system previously tested in CHO cells (Baaske et al., 2018)

**(E)** CRISPRi	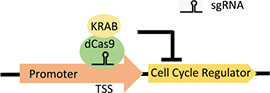	• No premodification of target required • Efficient downregulation of gene expression	• Slower acting cell-cycle arrest due to reliance on half-life of POI • Reliant on the position of gene in the genome for knockdown efficiency	• Not yet used to target cell-cycle regulators in CHO cells • Previously used to knockdown endogenous apoptotic genes in CHO cells (Xiong et al., 2019)

**(F)** CRISPRa	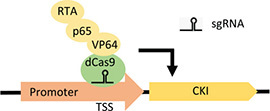	• Potentially higher fold increase in CKI gene expression	• Reliant on the position of gene in the genome for activation efficiency	• Not yet used to target cell-cycle regulators in CHO cells • Previously used to upregulate of endogenous genes in CHO cells (Karottki et al., 2020)

## Novel Synthetic Biology Methods for Proliferation Control

Since the original, cell engineering-based proliferation control studies (Fussenegger et al., 1997; Mazur et al., 1998; Carvalhal et al., 2003; Bi et al., 2004), multiple synthetic biology tools have emerged, including inducible gene expression systems (Kallunki et al., 2019), degradation systems (Trauth et al., 2019), and gene knockout/repression systems (Guha et al., 2017). However, the application of such tools in decoupling growth and production in mammalian cells has largely remained unexplored. There have been significant advances in synthetic biology approaches to decoupling growth and production in microbial systems, based on mRNA interferases (Suzuki et al., 2005), control of host RNA polymerase activity (Izard et al., 2015; Stargardt et al., 2020), and regulation of DNA replication machinery (Li et al., 2016). Recently, a bacteriophage-derived *Escherichia coli* RNA polymerase inhibitor was used to arrest cell growth by inhibiting σ-factor 70-driven host gene expression (Stargardt et al., 2020). Although mammalian proliferation control pathways are more complex, similar approaches could be used to target the mammalian cell cycle. The potential of synthetic biology in decoupling growth and production in mammalian cells has been partly realized in “purity by design” cell lines. Activation of a caffeine-inducible mammalian protein kinase R switches off non-product protein translation, while a viral IRES sequence protects recombinant protein production (Bojar et al., 2019). However, titer is limited because translation from IRES sequences does not have access to the full ribosomal machinery (Table 1B; Bojar et al., 2019). Although this method could be incredibly valuable for products where the presence of host cell proteins may be particularly harmful to product quality, it is inherently limited when improving product titer is a priority, as is common need for difficult-to-express proteins.

Degradation systems target endogenous cellular proteins and could be used to rapidly knock down cell-cycle regulator activity (Trauth et al., 2019). A degron, added to the N- or C-terminus of a protein of interest (POI), allows targeted and rapid protein degradation upon addition of a small-molecule inducer (Table 1C). An example of such a system is the Auxin-Inducible Degron (AID) system. AID-mediated degradation of RAD21-mAC rapidly arrested growth of human cells (Natsume et al., 2016). However, fusion of the endogenous POI with the degron tag requires considerable effort and, with the AID system, implementation of the strategy requires the expression of additional heterologous proteins (TIR1). Moreover, a degron-tagged cell-cycle regulator could negatively impact cell proliferation during the growth phase if protein activity is reduced. However, a degron-tagged topoisomerase IIα did not impact proliferation rate or culture viability, despite the enzyme’s essential role in the cell cycle (Farr et al., 2014). Imperative for the application of degron systems in industrial bioproduction is that the small-molecule inducer is non-toxic, physiologically inactive, and inexpensive (Weber and Fussenegger, 2007). However, metabolic bi-products of auxin have displayed toxicity in mammalian cell lines (Kim et al., 2004, 2010). Optogenetic approaches could overcome such limitations, since light is non-toxic and can be instantly applied and removed during culture. For example, the dual-controlled “Blue-OFF” system combines a KRAB-EL222 light-inducible repressor with a B-LID (blue light-inducible degradation domain) protein degradation module, to rapidly downregulate target protein levels in response to blue light (Baaske et al., 2018). By combining such systems with recently developed light-inducible gene expression tools (Yamada et al., 2020), blue light could be used to trigger both cell-cycle regulator degradation (to halt proliferation) and expression of the product gene (to start the production phase).

At the forefront of synthetic biology improvements has been the development of CRISPR/Cas9 as a tool for genetic manipulation (Boettcher and McManus, 2015). The sgRNA-guided Cas9 endonuclease generates double-stranded breaks in target-gene sequences, which are repaired by the error-prone non-homologous end-joining pathway, generating a variety of mutations (termed CRISPRn). CRISPR/Cas9 methods have already been extensively used for enhancing recombinant gene expression in CHO cell bioproduction (Dangi et al., 2018). A fusion of a catalytically inactive Cas9 (dCas9) and the Krüppel-associated box (KRAB) repression domain, targeted to a transcriptional start site (TSS), can be used to silence expression of a target gene (termed CRISPR interference or CRISPRi) (Table 1D). An inducible version of CRISPRi is created by controlling dCas9 or sgRNA expression with an inducible gene expression system. The application of CRISPRi for decoupling growth and production has already been realized in bacterial cells, where knockdown of DNA replication machinery or nucleotide synthesis was shown to cause cell arrest (Li et al., 2016). Using RNAi to knock down cyclin E1 and E2 expression, cell proliferation was arrested in the G1 phase in human hepatocellular carcinoma cell lines (Geng et al., 2018). This could be repeated with CRISPRi, a method that gives more consistent and robust gene knockdown than RNAi, while exhibiting less off-target effects (Boettcher and McManus, 2015). Alternatively, dCas9 can be used for the upregulation of target-gene expression (termed CRISPR activation or CRISPRa). The VPR CRISPRa approach requires the direct fusion of dCas9 with transcriptional activators (VP64, p65, and Rta), targeted to the TSS of endogenous genes with sgRNAs (Table 1E; Chavez et al., 2015). CRISPRa could be used to arrest proliferation by upregulating endogenous CKI gene expression (Table 1F). Although the degree of target-gene upregulation is highly loci dependent, CRISPRa allows for much higher fold increases in target-gene expression than what is common for standard inducible promoter systems (Chavez et al., 2015). This could overcome the problem of weak cell-cycle arrest in previous CKI-overexpression studies.

## Selection of Suitable Targets

Identification of cell-cycle targets for the purposes of cancer treatment has shown that the impact of knocking down cell-cycle regulators is often cell line specific. For example, *Cdk2* knockdown had no significant impact on cell proliferation in certain mammalian cell lines, whereas in other studies, *Cdk2* knockdown induced cell-cycle arrest (Tetsu and McCormick, 2003; Liu et al., 2020). Potential targets for knockdown or overexpression are outlined in Figure 1. Selection of certain cell-cycle regulators for gene knockdown/protein degradation may be limited by their interaction with apoptotic pathways. To minimize the risk of inducing apoptosis after knockdown of cell-cycle regulators, proliferation control strategies could be combined with overexpression or knockout of key apoptotic genes (Kim and Lee, 2000, 2002). Reducing apoptosis could also help maintain high cell densities during the production phase for a longer time period, thus improving product titer.

Transcriptional inhibition approaches, such as CRISPRi, are inherently slower acting than degradation methods, due to a reliance on the inherent half-life of the protein, meaning proteins with longer half-lives take longer to remove. Cell-cycle regulators with short half-lives, such as D-type cyclins (half-life of 30 min) (Donjerkovic and Scott, 2000), are likely to provide more suitable targets for transcription-based regulation. Challenges for implementing this strategy will also arise from the genetic plasticity that CHO cells exhibit, giving rise to modifications in gene copy number, changes in transcriptional activity, and substantial chromosome rearrangements (Vcelar et al., 2018). A mutation in the proliferation control pathway could lead to cells escaping arrest. Additionally, the gene knockdown strategy would have to arrest the majority of cells in the culture. The efficiency of CRISPRi-mediated knockdown is known to be largely dependent on the genomic context of the target gene, meaning that high knockdown efficiencies of cell-cycle regulators may not easily be achieved. If these challenges are not addressed, populations of fast-growing cells may outcompete the arrested cells, limiting culture duration and product titer. Targeting multiple proliferation pathways is a strategy that is common in cancer therapies and could reduce the number of cells escaping cell-cycle arrest. To improve CRISPRi efficiency, cell-cycle regulators could be targeted more easily by knocking out the endogenous gene and expressing a recombinant version of the gene at a more easily targeted locus, using a targeted integration system.

## Discussion

With the increasing demands on biomanufacturing facilities to produce a wider range of protein biotherapeutics at higher titers and in a shorter period of time, decoupling growth and production provides a viable alternative to traditional constitutive expression strategies. Moving the product expression window away from the growth phase mitigates the negative impact that cytotoxic proteins have on cell growth, viability, and stability. Additionally, there is increasing evidence that recombinant protein expression places a substantial metabolic burden on the cell, particularly on its secretory capacity, resulting in decreased cell line stability and downregulation of recombinant gene expression (Jones et al., 2005; Ong et al., 2019; Poulain et al., 2019). For example, removing the expression of a non-essential recombinant gene substantially improved the growth characteristics of a CHO cell line (Kallehauge et al., 2017). This suggests that decoupling growth and production could have wider application in the production of biotherapeutics, beyond just those that are cytotoxic.

The application of decoupling strategies in bioproduction has been limited by the variable impact of temperature shifts and chemical agents on product titer, culture viability, and product quality. This is largely due to the interaction of these strategies with many other cellular pathways that are not related to cell proliferation. Although industrially inviable due to their cost, the use of highly selective cancer drugs for proliferation control has yielded promising results for targeted approaches (Du et al., 2015). Synthetic biology offers a potentially more industrially relevant approach, but its use in proliferation control has remained largely unexplored. Here, we have discussed synthetic biology tools that have emerged over the last 20 years that could be used to arrest cell proliferation. Synthetic biology approaches to decoupling growth and production in microbial systems have been developed, but a mammalian cell engineering approach remains largely unexplored. The implementation of such strategies in mammalian cells has considerable advantages over conventional approaches to proliferation control, allowing the selective targeting of cell-cycle regulators to induce cell-cycle arrest (with the associated increase in specific productivity), while avoiding negative impacts on product quality.

## Data Availability Statement

The original contributions presented in the study are included in the article/supplementary material, further inquiries can be directed to the corresponding author/s.

## Author Contributions

JD and MD developed the presented idea. JD wrote the manuscript in consultation with SR and MD. All authors contributed to the article and approved the submitted version.

## Conflict of Interest

The authors declare that the research was conducted in the absence of any commercial or financial relationships that could be construed as a potential conflict of interest.
